# On the Study of Joint YOLOv5-DeepSort Detection and Tracking Algorithm for *Rhynchophorus ferrugineus*

**DOI:** 10.3390/insects16020219

**Published:** 2025-02-17

**Authors:** Shuai Wu, Jianping Wang, Wei Wei, Xiangchuan Ji, Bin Yang, Danyang Chen, Huimin Lu, Li Liu

**Affiliations:** 1 School of Computer and Communication Engineering, University of Science and Technology Beijing, Beijing 100083, China; 2 China Mobile Group Design Institute Co., Ltd., Beijing 100080, China; 3 Hainan Key Laboratory of Tropical Oil Crops Biology, Coconut Research Institute of Chinese Academy of Tropical Agricultural Sciences, Wenchang 571339, China

**Keywords:** Red Palm Weevil, YOLOv5, joint YOLOv5-DeepSort, target tracking

## Abstract

The Red Palm Weevil (RPW, *Rhynchophorus ferrugineus*) is a destructive pest of palm plants that can cause the death of the entire plant when infested. To enhance the efficiency of RPW control, a novel detection and tracking algorithm based on the joint YOLOv5-DeepSort algorithm is proposed. Firstly, the original YOLOv5 is improved by adding a small object detection layer and an attention mechanism. At the same time, the detector of the original DeepSort is changed to the improved YOLOv5. Then, a historical frame data module is introduced into DeepSort to reduce the number of target identity (ID) switches while maintaining detection and tracking accuracy. Finally, an experiment is conducted to evaluate the joint YOLOv5-DeepSort detection and tracking algorithm. The experimental results show that, in terms of detectors, the improved YOLOv5 model achieves a mean average precision (mAP@.5) of 90.1% and a precision (P) of 93.8%. In terms of tracking performance, the joint YOLOv5-DeepSort algorithm achieves a Multiple Object Tracking Accuracy (MOTA) of 94.3%, a Multiple Object Tracking Precision (MOTP) of 90.14%, reduces ID switches by 33.3%, and realizes a count accuracy of 94.1%. These results demonstrate that the improved algorithm meets the practical requirements for RPW field detection and tracking.

## Simple Summary

The Red Palm Weevil (RPW, *Rhynchhophorus ferrogineus*) is a destructive pest of palm plants. To improve the efficiency of RPW control, this paper proposes a detection and tracking algorithm based on the joint YOLOv5 (You Only Look Once version 5)-DeepSort (Deep Simple Online and Real-time Tracking) algorithm. This algorithm can detect and track pests, improving the efficiency of pest control. The experimental results show that the joint YOLOv5 DeepSort algorithm achieved a tracking accuracy of 94.3% and a counting accuracy of 94.1%, which can meet the practical requirements of RPW field detection and tracking.

## 1. Introduction

The Red Palm Weevil (RPW, *Rhynchophorus ferrugineus*) is a destructive pest of palms [1]. Infestations of the RPW have been observed in regions such as Hainan and Yunnan in China. The pest typically causes damage by its larvae feeding inside the heart and leaves of the trunk and crown of the tree, and this initial infestation is difficult to detect until later stages when it becomes apparent [2]. Once RPW has infested a plant, it will cause much of the plant to die. The pest will continue to spread and reproduce, potentially destroying entire plantations, unless effective control measures are taken [1]. Therefore, integrated management measures based on prevention should be given preference in the management of such pests. By manually monitoring the canopy for abnormal changes, such as finding fiber debris at the top of the canopy or hearing the sound of infestation inside the trunk, appropriate control measures can be taken immediately. However, manual control is costly and inefficient. Early detection of RPW infestation has been a major research focus and challenge for effectively protecting palm trees. In recent years, with the continuous development of artificial intelligence technology, deep learning and image recognition technologies have demonstrated great potential in pest detection. Applying these emerging technologies opens up new ways of detecting pests, which can accurately identify the type of pest before large infestations occur and quickly take measures to control the spread of pests [3]. Therefore, it is of great importance for pest control to investigate an efficient and accurate intelligent RPW detection system by introducing artificial intelligence.

The traditional control measure of RPW mainly relies on pheromone trapping and manual counting, which causes a waste of manpower and material resources and fails to meet the demand for intelligent pest prevention. In recent years, target detection technology has been widely applied in various fields with the rapid development of image processing and deep learning technology. Deep learning-based target detection technology has been widely used in plant pest identification. Common algorithms for target detection can be divided into two categories: two-stage and one-stage detection algorithms. Two-stage algorithms, represented by RCNN [4], e.g., R-CNN, Fast R-CNN [5], and Faster R-CNN [6], first generate a set of candidate boxes before employing a convolutional network to identify the detected targets. These algorithms achieve high detection accuracy but are slower. One-stage algorithms, represented by SSD [7,8] and the YOLO series [9], directly generate the class probability and location information of targets in a single step, providing the final detection results more rapidly, but with a loss of accuracy compared to two-stage algorithms. Among the single-step detection algorithms, SSD neural network runs slower than YOLO. The detection accuracy is slightly lower than that of Faster CNN, and the stability is also worse. In the YOLO series, YOLOv5 is one of the more advanced algorithms at present, with the advantages of fast detection speed on average, strong flexibility, and rapid deployment, which can be widely applied to the rapid and real-time detection of the RPW in natural and complex environments [10].

Post-identification counting is a key step in quickly and efficiently counting insect densities and implementing control measures. However, the RPW is often moving in the field environment, and there is a high degree of similarity between individual insects. Therefore, real-time tracking and counting cannot be achieved by a single target detection algorithm. The target tracking algorithm is able to track the trajectory of the RPW in real-time, providing the basis for an accurate count. Target tracking is a complex process that relies on contextual information to predict the behaviors and trajectories of the target, and then achieve continuous tracking of the target [11]. Wax [12] started the field’s development by first elaborating on the basic theory of target tracking. Chan et al. [13] introduced Kalman filtering into the tracking algorithm, which had a profound impact on progress [14]. In the fields of surveillance and driving, target tracking has been widely used as a popular research direction [15]. Currently, common target tracking can be divided into single-object tracking and multi-object tracking [16]. Single-object tracking [17] is the process of selecting only one target in the first frame of a video sequence and tracking that target continually. Multi-object tracking [18] is the process of simultaneously tracking the trajectories of multiple targets in a video sequence, which has a higher research value due to the closer proximity of the objects compared to single-object tracking. Common multi-object tracking algorithms are the SORT (Simple Online and Real-time Tracking) [19] algorithm and its enhanced version, the DeepSort (Simple Online and Real-time Tracking with Deep association metric) [20] algorithm. SORT is a real-time and efficient multi-object tracking algorithm capable of accurately tracking multiple targets in complex environments [21]. It essentially consists of a detector and a tracker. The detector relies on advanced deep learning models to perform the target detection task, while the tracker tracks the targets in real-time based on the recognition results. This architecture enables the SORT algorithm to perform multi-target tracking tasks in complex scenes accurately and efficiently. However, during the target tracking process, the SORT algorithm suffers from the problem of ID switching due to target occlusion. The DeepSort algorithm is an improvement of the SORT algorithm designed to solve the ID switching problem in scenarios [22], and the algorithm introduces the cascade matching technique, further improving tracking efficiency.

In this paper, considering the requirements of tracking and counting tasks in RPW control, a joint YOLOv5-DeepSort detection and tracking algorithm is proposed. It is improved and optimized based on the original DeepSort multi-object tracking algorithm. Firstly, given the small target characteristics of RPW, the detector part of DeepSort is replaced by an improved YOLOv5 model that incorporates a small target detection layer and attention mechanism to enhance the detection accuracy significantly. In addition, to improve tracking accuracy and counting precision, a historical frame data module is integrated into the trajectory processing part of DeepSort, effectively reducing identity switches during tracking. Comparative experiments are then performed on a self-generated RPW dataset, and the results are evaluated using commonly used evaluation metrics. Finally, the experimental results demonstrate that the proposed joint algorithm achieves high precision in detecting and tracking RPWs.

The rest of the paper is structured as follows: Section 2 describes the experimental and dataset settings. Section 3 describes the principles of the relevant algorithms. Section 4 presents the experiment and the analysis of the results. Section 5 provides a summary of the work.

## 2. Materials and Methods

### 2.1. Dataset Acquisition and Labeling

**Figure 1:**
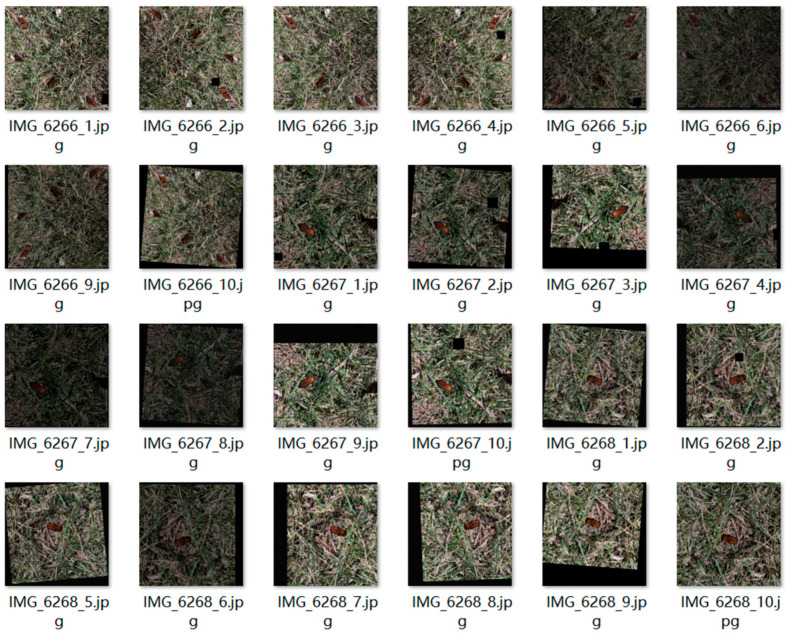
Partial presentation of the PRW dataset (image dataset for object detection).

This paper takes RPW as the research object; home-made datasets are adopted by taking RPW-related images and producing RPW target detection datasets and target tracking datasets on their own. The shooting device is the rear camera of the mobile phone, and the image resolution is 3024 pixels × 3024 pixels. The shooting environment is outdoor. After shooting, the images are screened to filter out the blurred target images [23]. Finally, 305 images of RPW with different shooting angles and scenes are obtained. A total of 2600 RPW images are obtained through data augmentation and other operations. This target detection dataset is divided into the training, validation, and test sets in a ratio of 7:2:1, resulting in 1820 images in the training set, 520 images in the validation set, and 260 images in the test set. A partial representation of the PRW dataset is shown in Figure 1.

**Figure 2:**
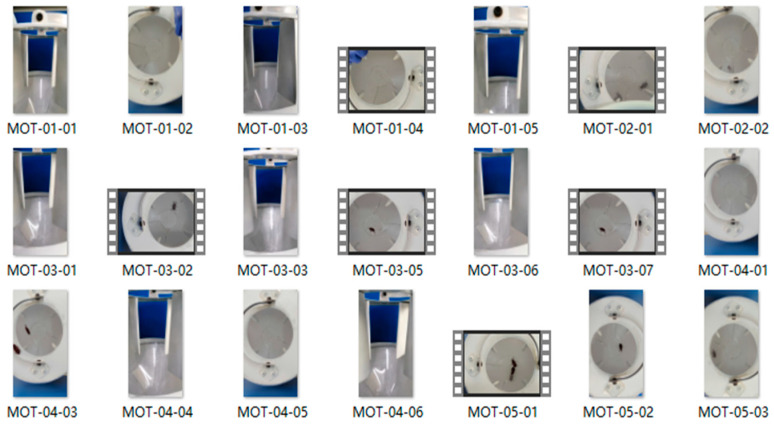
Partial presentation of the PRW tracking test dataset (derived from video frame data).

The target tracking classification dataset is produced based on the target detection dataset. Firstly, the target RPWs are extracted from the 2600 images in the object detection dataset, resulting in a target tracking classification dataset comprising 4272 images. Secondly, the target tracking classification dataset is divided into training and validation sets, totaling 85 categories. In subsequent procedures, 21 target tracking test video sets are constructed based on the MOT-16 format to validate the effectiveness of the algorithm. The video data are recorded using smartphones, with the subjects being RPWs trapped under laboratory conditions. The average frame rate of all videos is maintained at 30 frames per second, ensuring that the video quality fulfills the requirements for analysis. A partial presentation of the target tracking test dataset is shown in Figure 2.

**Figure 3:**
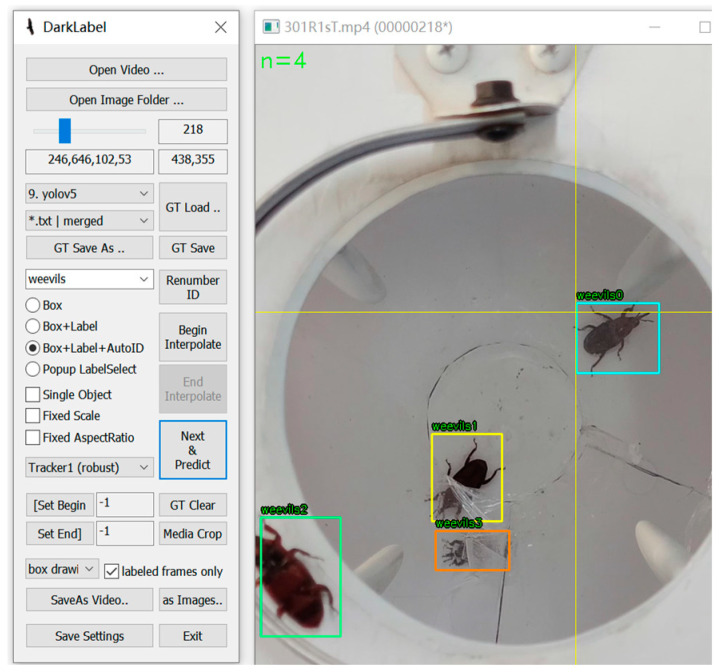
Diagram of the labeling process for the target tracking test dataset.

The target tracking dataset is labelled using DarkLabel software version 2.4. The software has rich functions and supports a variety of annotation formats for the target tracking dataset, including rectangular boxes, polygons, keypoints, etc. [24]. By using DarkLabel, the task of labeling the target tracking dataset can be performed efficiently, and the process of labeling the dataset is shown in Figure 3.

### 2.2. Data Augmentation

**Figure 4:**
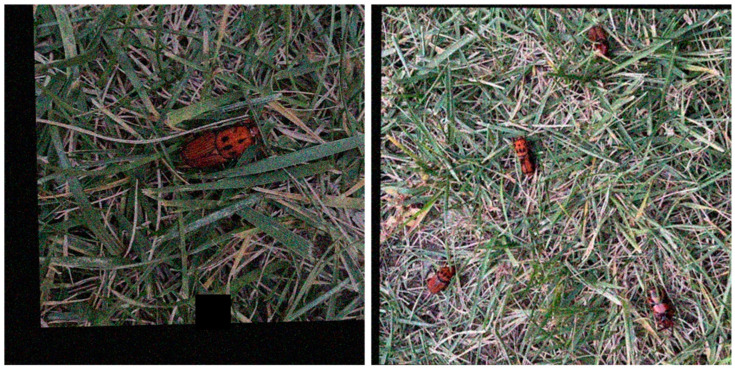
Schematic of selected samples after data augmentation (only partial images are shown).

After data annotation, data augmentation processing is applied to the original data to improve the generalization performance of the model. A variety of common data augmentation methods are adopted, including the following: (1) Flip: The image is randomly flipped from 0° to 180° angle range [25]. (2) Noise addition: Different forms of noise, such as pretzel noise and Gaussian noise, are introduced to simulate the complexity in the actual scene [25]. (3) Brightness and contrast change: The image’s brightness and contrast are adjusted to increase the degree of variation [26]. These data augmentation methods finally obtained 2600 images of RPW samples. The above data augmentation helps improve the model’s generalization ability and adaptability to complex scenes, and expands the dataset. Figure 4 shows some of the images processed by data augmentation, and such processing not only enriches the dataset but also helps the model to better learn and generalize to new scenes.

## 3. Detecting and Tracking Principles

A joint YOLOv5-DeepSort algorithm is proposed for the efficient control of RPWs and the elimination of the drawbacks of traditional control methods. An improved YOLOv5 model for detection and identification is followed by the optimized DeepSort algorithm for tracking and counting. The joint algorithm incorporates historical data information during target movement to accurately track and count the RPWs, ultimately enabling intelligent prediction of RPWs.

### 3.1. Improved YOLOv5 Model

**Figure 5:**
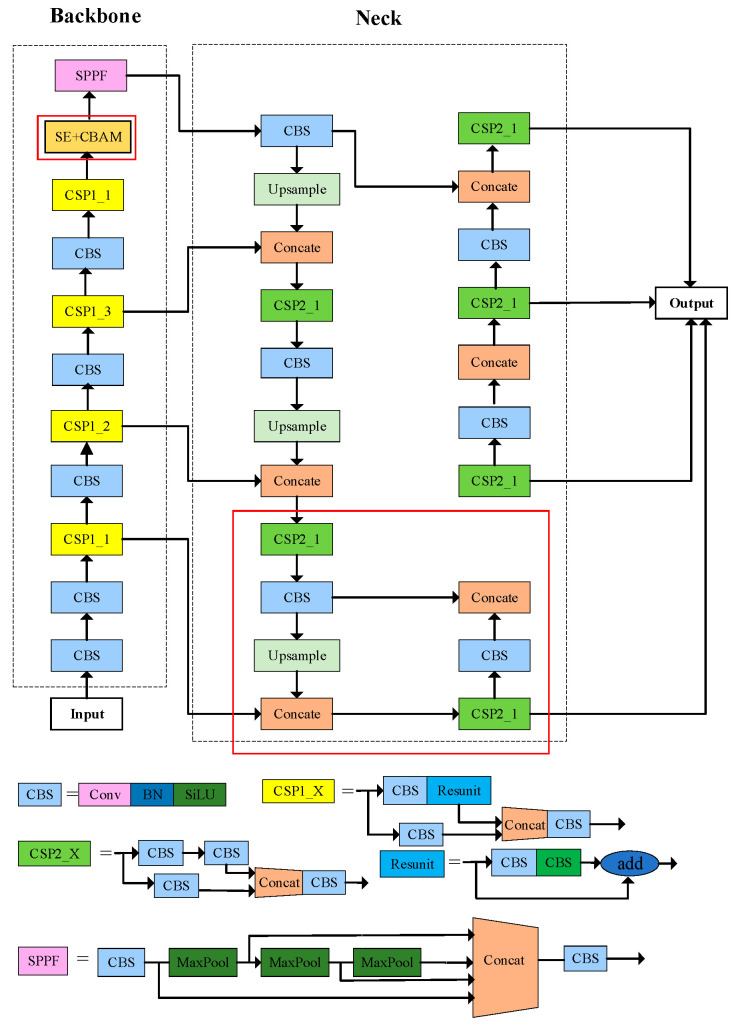
Improved YOLOv5 network architecture.

Target detection is the basis of tracking and counting algorithms. In this work, the improved YOLOv5 is adopted as the detector part of DeepSort. Currently, YOLOv5 is one of the more advanced and well-established target detection algorithms, widely used due to its advantages of fast average detection speed and rapid deployability. Its network structure consists of input, backbone network, neck network, and output, which can be applied to the fast real-time detection of RPWs in complex environments [26]. However, the target of the RPW in the field environment is too small, and there are more similar insects and complex backgrounds, which affects the detection performance of the detection algorithm for this pest. To solve the above problems, the network structure of the original YOLOv5 model is improved [27]. The specific improvements are as follows:

(1) Improving the feature extraction network: The difficulty of feature extraction is increased because the RPW as a small target occupies a small proportion of the whole background. To address the above problems, a 4-fold down-sampling layer is added in the feature extraction network to improve the ability to extract the location information of small targets.
(2) Fusion attention mechanism: For the problem of the RPW being difficult to detect when features are missing, Squeeze-and-Excitation (SE) [28] and Convolutional Block Attention Module (CBAM) [29] attention mechanisms are added to the original YOLOv5. These mechanisms enhance the algorithm’s local feature extraction ability and improve the detection performance. The improved YOLOv5 network is given in Figure 5, and the improved part of the network is shown in the red box.

### 3.2. DeepSort Target Tracking Algorithm

**Figure 6:**
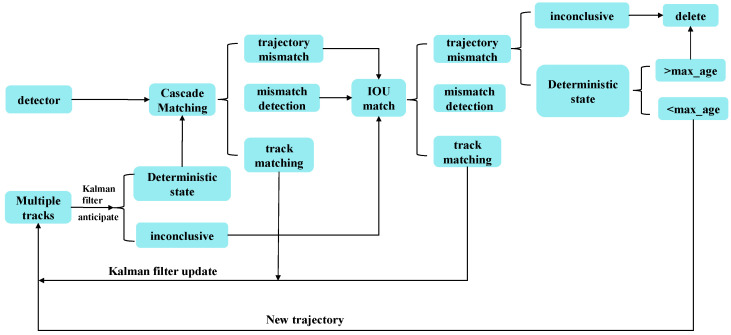
The flowchart of the original DeepSort.

The DeepSort algorithm [20] is an improved version of the SORT algorithm that effectively solves the problem of frequent identity (ID) switching by introducing a more stable CNN-based metric and training on large datasets [30]. The flowchart of the algorithm is presented in Figure 6, and the main workflow of DeepSort is as follows [31].

Step 1: A sequence of video frames is input for tracking. Whether the sequence reaches the endpoint is checked, and, if so, the task is marked as complete; otherwise, the target detector is applied to the current frame.

Step 2: The detected frame is the intersection over union (IOU) with the predicted frame, and the cost matrix is generated based on these matching results.

Step 3: The cost matrix is linearly matched using the Hungarian algorithm, and the possible matching results are classified into three categories:

(1) Trajectory mismatch: If a prediction frame cannot be matched with any detection frame, it may mean that the target has left the field of view, and therefore the ID of that target must be removed;

(2) Detection mismatch: If a detection frame exists but no corresponding trajectory match is found, this indicates that a new target has appeared, in which case a new trajectory will be created for that target;

(3) Trajectory matching: If the IOU match between the detection frame and the prediction frame meets the requirements, Kalman filtering will immediately update the prediction frame, and, if this process continues to meet the matching conditions, tracking will continue.

Step 4: Step 2 and Step 3 are repeated until the trajectory is confirmed or the video sequence ends.

Step 5: Kalman filtering is adopted to predict the bounding boxes of the confirmed and unconfirmed state trajectories.

Step 6: The inputs to the Hungarian algorithm are replaced by a cost matrix to produce matching results.

Step 7: The operations of step 5 and step 6 are repeated until the end of the video frame, which terminates the loop.

### 3.3. Improved DeepSort Algorithm

**Figure 7:**
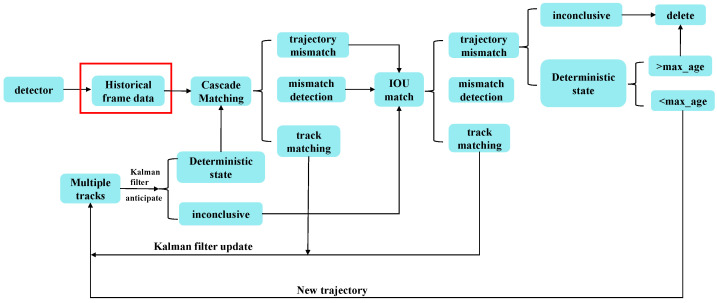
The flowchart of the improved DeepSort algorithm (the main improvement here is the addition of a historical frame data module, as shown in the red box).

The high similarity between individual RPWs complicates the model’s ability to distinguish them during tracking. In addition, the trajectory of the RPWs lacks regularity. Their sudden take-off and landing behavior leads to drastic changes in speed and direction, which increases the complexity of tracking. Furthermore, partial occlusion and cross-motion between targets further exacerbate the tracking difficulty in a scene where multiple targets are moving at the same time. All these factors lead to the ID-hopping problem in the DeepSort algorithm, which has an impact on its tracking performance. ID hopping refers to the fact that during the target tracking process, the unique identity of the target changes to some extent, resulting in reduced tracking accuracy. Therefore, by introducing the historical frame data module, the DeepSort algorithm is improved and optimized to deal with the ID hopping problem and improve tracking accuracy. The improved DeepSort algorithm is shown in Figure 7, where the part in the red box is the improved module. The main workflow is as follows:

Step 1: A new module Target_dict is introduced in the trajectory information processing part of the DeepSort network, which is used to save the historical trajectory information of the RPWs, as shown in the red rectangular box in Figure 7. This module is indexed by the ID of the target, and its value contains information such as the target’s historical position, frame index, etc. By using global variables, it is possible to share and update the historical information of the RPWs between different frames.

Step 2: The target location information function is modified and the history information is updated. As described in the cascade matching and IOU matching in Figure 7, during target detection and tracking in each frame, whether the ID of the target individual exists in Target_dict is checked. If it exists, the reasonableness of the target ID is verified and the historical information of the target is updated; otherwise, a new ID is reassigned.

Step 3: The validation logic module continues to be introduced in the trajectory processing module to check the reasonableness of the target ID, as described in the Kalman filter update step for multiple trajectories in Figure 7. The distance and speed information between the insects is used to judge the reasonableness of the ID in the RPW tracking scenario. Simultaneously, the new ID is set to the maximum of the current ID plus one to prevent unreasonable IDs.

### 3.4. The Joint YOLOv5-DeepSort Algorithm

**Figure 8:**
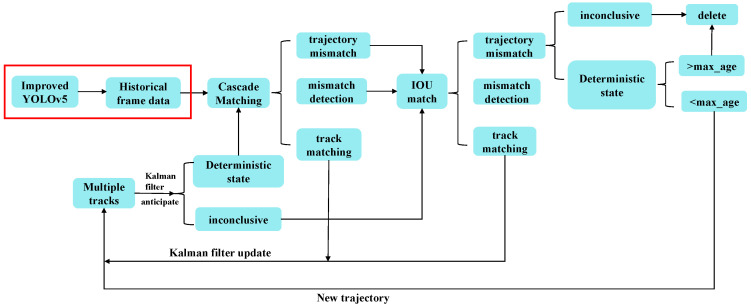
The flowchart of the joint YOLOv5-DeepSort architecture.

Based on the above research, the improved YOLOv5 model is combined with the DeepSort target tracking algorithm, which fuses historical image data, to form a joint YOLOv5-DeepSort algorithm. The algorithm can achieve high-accuracy detection of small targets such as RPWs in the field environment. It also has a good detection effect in the case of feature occlusion. In addition, the joint algorithm can be adapted to real-time monitoring and tracking of moving targets, and has good tracking performance for insects with highly similar features. The specific workflow of this joint algorithm is shown in Figure 8, where the red box is the optimization part of this work.

## 4. Experiment and Results Analysis

### 4.1. Experimental Equipment and Parameter Settings

The deep learning framework is PyTorch, running on Windows 10. The hardware platform uses the NVIDIA GeForce GTX 1070 graphics card, with 8 GB of video memory capacity. CUDA version 10.2 is used for parallel computing, with 1000 iterations. For the specific parameter settings for model training, the following configurations are used.

(1) Learning rate: set to 0.01, which is used to control the step size of the model to update the weights in each training iteration.
(2) Momentum: set to 0.937, this parameter helps to speed up the convergence process and reduce the oscillation of the training process.
(3) Decay: set to 0.0005, which is used to control the decay rate of the learning rate to maintain the stability of the training.
(4) Batch size: set to 8, indicates the number of samples processed simultaneously in each iteration.

### 4.2. Evaluation Metrics for the Red Palm Weevil Dataset

#### 4.2.1. Target Detection Dataset Evaluation Indicators

Several indicators are adopted to evaluate the experimental training results of the RPW to ensure the accuracy of the experimental results. The evaluation indicators in the field of common target detection are Precision (P), Recall (R), F1—score, Average Precision (AP), Mean Average Precision (mAP), Intersection over Union (IoU), FPS (frames per second), etc. [32]. FPS represents the number of frames per second that can be processed by the object detection method, used to verify the real-time performance of the detection method. The above indicators can be represented by the following calculation process:

(1) $$P=\frac{TP}{TP+FP}$$

(2) $$R=\frac{TP}{TP+FN}$$

(3) $$AP=\int_{0}^{1}P(R)dR$$

(4) $$mAP=\frac{\sum_{i=1}^{N}AP_{i}}{N}$$

(5) $$IoU=\frac{B_{a}\cap B_{b}}{B_{a}\cup B_{b}}$$

where *TP* denotes the number of positive classes detected as positive classes, *FP* denotes the number of negative classes detected as positive classes, *FN* denotes the number of positive classes detected as negative classes, and *N* is the number of detected sample classes. The F1-score is the harmonic mean of precision and recall, used for comprehensively evaluating the performance of classification models. *B_a_* denotes the region of the prediction frame and *B_b_* denotes the region of the real frame. The intersection and merger ratio IoU represents the degree of overlap between the real frame and the predicted frame, and a higher IoU value indicates a more accurate result. When the IoU threshold is set to 0.5, the mAP at this time is denoted as mAP@.5. Setting the threshold range of IoU to 0.5~0.95, with a step size of 0.05, results in a total of 10 different thresholds. Then, the corresponding mAP and the average mAP@.5:.95 are calculated. Since the detected P and mAP more accurately reflect the prediction accuracy of the general model, the subsequent analysis will focus on P and mAP [33].

#### 4.2.2. Target Tracking Dataset Evaluation Indicators

To verify the effectiveness of the target tracking algorithm, the following common indicators of the target tracking algorithm are selected to evaluate tracking performance: Multiple Object Tracking Accuracy (MOTA), Multiple Object Tracking Precision (MOTP), the total number of incorrect changes occurring in the change of identity (IDS), and counting performance [34]. The MOTA and MOTP are described as follows:

MOTA denotes the accuracy of the multi-object tracking algorithm and its formula is shown in Equation (6), where *FN* denotes the number of missed detections, *FP* denotes the number of false detections, and IDS denotes the number of tracking target identity changes. This index comprehensively considers the effects of leakage detection rate, misdetection rate, and ID switching rate on the tracking accuracy.

(6) $$MOTA=1-\frac{\sum_{t}\left(FN_{t}+FP_{t}+IDS\right)}{\sum_{t}GT_{t}}$$

MOTP denotes the degree of overlap between the predicted frame and the real frame, i.e., the target tracking algorithm precision, which is formulated in Equation (7), where *d_t,i_* denotes the average metric distance between the tracked predicted target *i* and its corresponding real frame over all frames, and *c_t_* denotes the number of correct matches between the target and the real frame at frame *t*.

(7) $$MOTP=\frac{\sum_{t,i}d_{t,i}}{\sum_{t}c_{t}}$$

### 4.3. Experiments and Analysis of Results

#### 4.3.1. Improved YOLOv5 Experiments and Results Analysis

To verify the reasonableness of the algorithm, this section conducts ablation experiments on the RPW dataset for the improvement effect of different modules. Here, √ indicates that the module is introduced into the original YOLOv5 model; × indicates that it is not introduced. The results of the ablation experiments are shown in Table 1, where 4× represents the introduction of four-fold down-sampling.

The improved YOLOv5 model proposed in this work is denoted as YOLOv5-Ours. Comparative experiments are conducted between YOLOv5-Ours and the original YOLOv5 to record the differences between the two models regarding P, mAP, etc. The results of the comparison experiments are shown in Table 2. To ensure the rigor of the experiments, the parameter settings and experimental platforms of all the above experiments are kept consistent. The comparison results are based on the detection data of the pre-improvement model, while the P, F1-score, and mAP@.5 are used as reference indicators. It is generally assumed that the higher the values of P, F1-score and mAP@.5, the better the trained weights and the better the model detection performance.

The results of the ablation experiments in Table 1 show that, compared with the original YOLOv5 model, the addition of the small target detection layer in the backbone network improves both the detection P and mAP. The P improves by 1%, mAP@.5 improves by 0.5%, and although there is an increase in these indicators, the improvement is not significant. With the addition of the SE attention mechanism, the P improves by 1.6%, and there is a decrease in both mean average precision (mAP) and detection effectiveness. Adding CBAM attention to the model improves P by 2%, but mAP still decreases. Furthermore, it can be observed that the introduction of any single module will result in a decrease in the F1-score. As can be seen from the results in Table 2, the introduction of the four-fold down-sampling layer and the SE and CBAM attention mechanisms leads to an observable improvement in P, F1-score and mAP for YOLOv5-Ours. The detection P is improved by 2.7% compared to the original YOLOv5, the F1-score increases by 1.7%, and there is also an improvement in the mAP: mAP@.5 improves by 1.4% and mAP@.5:.95 improves by 0.8%. Experimental validation further confirms the important role of the improved algorithm in enhancing performance.

**Figure 9:**
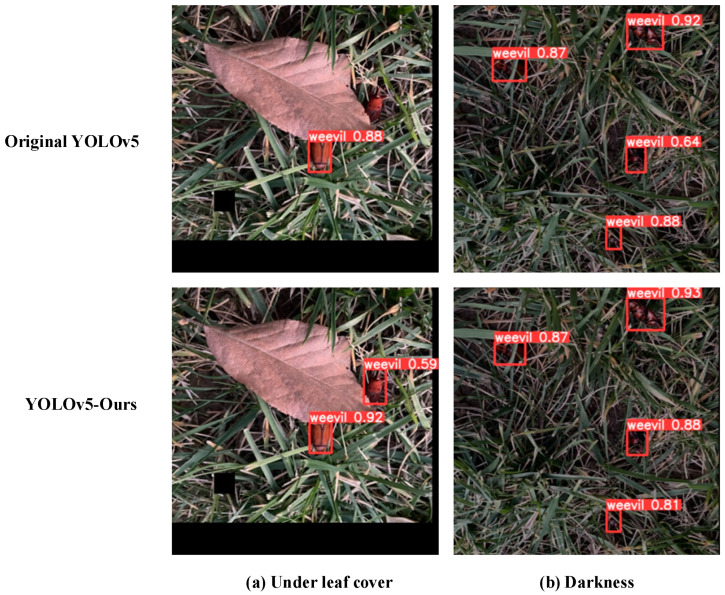
Detection performance results of different models when features are missing.

To intuitively demonstrate the detection performance of the improved model, the target detection tests are performed using the original YOLOv5 model and the YOLOv5-Ours model, respectively, by selecting the data under a number of complex scenes in the test set. The test results are shown in Figure 9. The detection performance when the sample is occluded and some features are missing is shown in Figure 9a, while the detection performance of the two models at night is shown in Figure 9b. As shown in Figure 9a, the original model is unable to identify the occluded RPWs in the absence of some sample features, while the improved model is able to detect the sample successfully at 92% accuracy. Figure 9b shows that, for the night scene, the detection accuracy of the improved model reaches 93%, which is significantly better than the original model.

#### 4.3.2. The Joint YOLOv5-DeepSort Performance Analysis

To verify the effectiveness of the DeepSort target tracking algorithm, comparative experiments are performed on the model before and after the improvement. The test results of the original DeepSort and the joint YOLOv5-DeepSort on the home-made tracking dataset are presented in Table 3. To ensure the rigor of the experiments, all parameter settings of the comparison experiments and the experimental platforms are kept the same. The evaluation results are measured against the experimental data of the original DeepSort algorithm. It can be seen that the joint YOLOv5-DeepSort algorithm improves the target tracking accuracy MOTA by 0.2% and the target tracking accuracy MOTP by 0.1% compared to the original algorithm from the experimental comparison results in Table 3. In addition, there is a 33.3% reduction in the number of ID switches. The experiments show that the joint algorithm improves all of the above target tracking evaluation indicators. It can be adapted to the practical tracking task of RPWs, thus laying the foundation for subsequent counting work.

**Figure 10:**
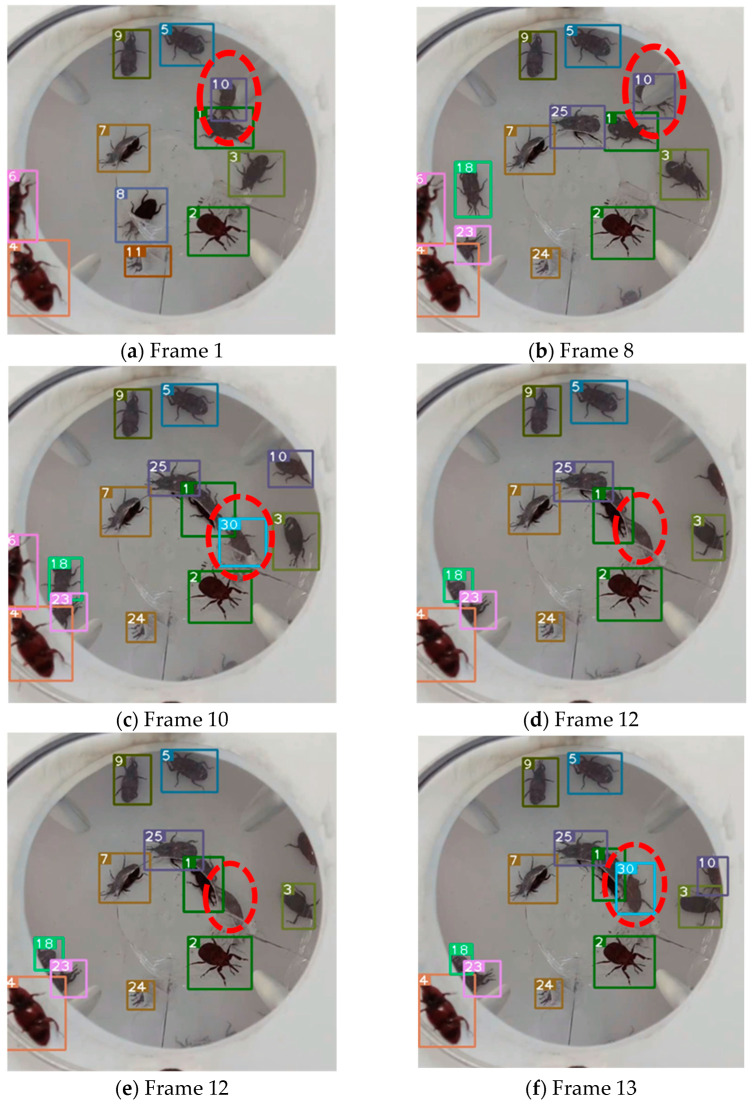

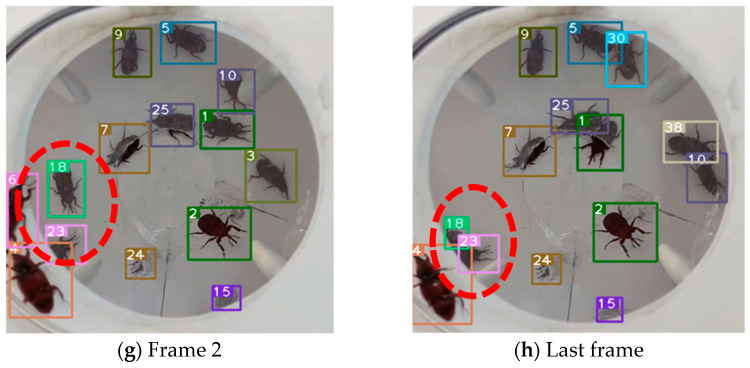
Tracking performance of improved DeepSort algorithm.

A visual analysis, shown in Figure 10, is conducted using several frames from a video in the RPW target tracking dataset to show the tracking performance more intuitively. As shown in Figure 10a,b, the RPW with ID 10 appears for the first time in frame 1, when it is not occluded, and in frame 8, most of the features of the RPW with ID 10 are occluded, but it is still tracked correctly without changing its ID. As illustrated in Figure 10c–e, the RPW with ID 30 appears for the first time in frame 10. When it moves to frame 12, it loses its identity due to the similarity and occlusion with the RPW with ID 1. However, the RPW with ID 30 is successfully tracked again at frame 13. As also shown in Figure 10f–h, the RPWs with IDs 18 and 23, which first appear in frame 2, are successfully tracked until the last frame of the video, although the features are mostly occluded. These cases demonstrate the effectiveness of the improved algorithm, which can be applied to real-time monitoring and tracking of RPW in realistic scenarios. Despite the loss of ID when the target is occluded, it can continue to be tracked very quickly, with most targets being correctly identified and fewer ID switches.

A novel tracking and counting method using the joint YOLOv5-DeepSort algorithm is used for the RPW counting task. To verify the effectiveness of the method, a total of 17 RPWs are randomly selected and compared with the manual counting method and the anchor frame counting method. The experimental results in Table 4 show that the joint YOLOv5-DeepSort algorithm in this work has a better counting performance, with 94.1% counting accuracy, higher than the anchor frame counting method introduced in the previous section.

## 5. Conclusions

To improve the control efficiency of the RPW, a joint YOLOv5-DeepSort algorithm is proposed for tracking and counting this pest. As a first step, the backbone of the original YOLOv5 model is improved. A small target detection layer is added to enhance the ability of the model to detect small targets, in response to the problem that the original YOLOv5 cannot detect small targets very well. Secondly, an attention mechanism is added to the network to enhance its ability to detect the target of the current task. Next, an improved DeepSort algorithm is introduced to address the problem of poor comfort with the traditional manual counting method. The original DeepSort algorithm is optimized to reduce ID hopping during the tracking process by incorporating a historical frame data module. In addition, the improved YOLOv5 model is combined with the optimized DeepSort, and the detector part of the optimized DeepSort is updated to YOLOv5-Ours. Finally, comparative experiments are performed on the joint algorithm. The results show that the precision of the joint YOLOv5-DeepSort algorithm reaches 93.8%, map@.5 reaches 90.1%, MOTA reaches 94.3%, MOTP reaches 90.14%, and the counting accuracy reaches 94.1%, meaning this algorithm can achieve high-precision real-time monitoring and counting of RPWs.

This work proposes a deeplearning-based monitoring technique for RPW. Although some research results have been achieved, there is still some work that can be carried out in the future. Based on the research in this article, several directions that can be carried out in future research work are proposed:

(1) Expand the sample dataset. The model proposed in this work demonstrates strong versatility in the fields of object detection and tracking, possessing the potential to be extended to applications for detecting and counting other pests. To enhance the applicability of the algorithm in other pest management systems, this work will consider further expanding the dataset by collecting more raw data from similar insects and covering more species and targets. Given the high similarity between the RPW and other insects, the current model may not be able to classify and detect these insects accurately. Therefore, further research is needed to integrate information from other modalities to improve the accuracy of insect classification and detection.
(2) Continuously improve model performance. In the future, we will continue to use deep learning models to train and optimize the enhanced data to improve the algorithm’s performance in recognizing species with similar shapes, especially on small target species such as insects that have similar appearances but less obvious features. At the same time, considering the limited number of individuals, the single scenario, and the limited types of target in the testing experiments in this article, the applicability of the algorithm can be expanded in the future by conducting hardware system experiments using the control variable method to improve the practical performance of the system.
(3) Research on model lightweighting. This work used optimized algorithm fusion to monitor and count the RPW, but the fused joint model had a relatively large volume, resulting in an overall increase in computational complexity. Considering the importance of model lightweighting for practical applications, in-depth research and discussion can be conducted in the future to reduce the complexity and storage requirements of models while maintaining computational performance. By using lightweight fusion models, more valuable references and guidance can be provided for the practical application of monitoring and counting models for RPW.

## Figures and Tables

**Table 1:** Results of ablation experiments on different modules.

4×	SE	CBAM	P	R	F1	mAP@.5	mAP@.5:.95	FPS
×	×	×	0.913	0.828	0.868	0.888	0.485	125
√	×	×	0.923	0.813	0.865	0.893	0.486	123
×	√	×	0.928	0.811	0.866	0.878	0.454	121
×	×	√	0.932	0.795	0.858	0.849	0.437	122
√	√	√	**0.938**	**0.834**	**0.883**	**0.901**	**0.489**	**120**

**Table 2:** Comparative experimental results of models before and after improvement.

Model	P	R	F1	mAP@.5	mAP@.5:.95	FPS
YOLOv5	0.913	0.828	0.868	0.888	0.485	125
YOLOv5-Ours	0.938	0.834	0.883	0.901	0.489	120

**Table 3:** Comparison of tracking results.

Algorithm	MOTA	MOTP	IDS
Original DeepSort	94.1%	90.03%	15
Joint YOLOv5-DeepSort	94.3%	90.14%	10

**Table 4:** Comparison of counting results.

Method	Result
Manual	17
Anchor Frame	19
Joint YOLOv5-DeepSort	16

## Data Availability

Relevant data are available from the authors upon reasonable request.
